# Non-recovery from dialysis-requiring acute kidney injury and short-term mortality and cardiovascular risk: a cohort study

**DOI:** 10.1186/s12882-018-0924-3

**Published:** 2018-06-11

**Authors:** Benjamin J. Lee, Chi-yuan Hsu, Rishi V. Parikh, Thomas K. Leong, Thida C. Tan, Sophia Walia, Kathleen D. Liu, Raymond K. Hsu, Alan S. Go

**Affiliations:** 1 Division of Nephrology, Department of Medicine, University of California, San Francisco, San Francisco, CA 94143 USA; 2 Division of Research, Kaiser Permanente Northern California, Oakland, CA 94612 USA; 3 Department of Epidemiology and Biostatistics, University of California, San Francisco, San Francisco, CA 94158 USA; 4 Division of Critical Care, Department of Anesthesia, University of California, San Francisco, San Francisco, CA 94143 USA

**Keywords:** Cardiovascular events, Mortality, End-stage renal disease, Dialysis-requiring acute kidney injury, Renal recovery

## Abstract

**Background:**

The high mortality and cardiovascular disease (CVD) burden in patients with end-stage renal disease (ESRD) is well-documented. Recent literature suggests that acute kidney injury is also associated with CVD. It is unknown whether patients with incident ESRD due to dialysis-requiring acute kidney injury (AKI-D) are at higher short-term risk for death and CVD events, compared with incident ESRD patients without preceding AKI-D. Few studies have examined the impact of recovery from AKI-D on subsequent CVD risk.

**Methods:**

In this retrospective cohort study, we evaluated adult members of Kaiser Permanente Northern California who initiated dialysis from January 2009 to September 2015. Preceding AKI-D and subsequent outcomes of death and CVD events (acute coronary syndrome, heart failure, ischemic stroke or transient ischemic attack) were identified from electronic health records. We performed multivariable Cox regression models adjusting for demographics, comorbidities, medication use, and laboratory results.

**Results:**

Compared to incident ESRD patients who experienced AKI-D (*n* = 1865), patients with ESRD not due to AKI-D (*n* = 3772) had significantly lower adjusted rates of death (adjusted hazard ratio [aHR] 0.56, 95% CI: 0.47–0.67) and heart failure hospitalization (aHR 0.45, 0.30–0.70). Compared to AKI-D patients who did not recover and progressed to ESRD, AKI-D patients who recovered (*n* = 1347) had a 30% lower adjusted relative rate of death (aHR 0.70, 0.55–0.88).

**Conclusions:**

Patients who transition to ESRD via AKI-D are a high-risk subgroup that may benefit from aggressive monitoring and medical management, particularly for heart failure. Recovery from AKI-D is independently associated with lower short-term mortality.

## Background

Patients with end-stage renal disease (ESRD) experience a high burden of early death and cardiovascular disease (CVD), particularly within the first year after dialysis initiation (189 to 382 per 1000 person-years) [1–7]. Furthermore, CVD contributes to > 50% of all ESRD deaths with known causes [8].

Recent literature suggests that acute kidney injury may also be associated with increased risk of CVD events [9–16]. Acute kidney injury is associated with increased inflammatory cytokines [17], which may increase risk of subsequent CVD events [18] via endothelial dysfunction or plaque rupture [19]. Of note, abrupt decline in kidney function during the three months before initiating hemodialysis is a strong, independent risk factor for early death after ESRD onset [20]. Dialysis-requiring acute kidney injury (AKI-D) is not an uncommon precipitant to ESRD [21, 22] and may be an important contributor to the high early mortality rates and excess CVD risk in incident ESRD patients. However, few studies have examined outcomes in this ESRD subgroup.

In a diverse, community-based cohort, we examined the association between AKI-D and short-term death and CVD outcomes. We hypothesized that patients with incident ESRD due to AKI-D are at higher short-term risk for death and CVD events compared to patients with incident ESRD that is not attributable to AKI-D, even after controlling for known vascular risk factors. Furthermore, we hypothesized that patients who recover adequate kidney function after AKI-D to discontinue dialysis would experience better outcomes. Our goal was to provide insight into the clinical implications of AKI-D as well as recovery from AKI-D to improve risk stratification and guide development of more tailored preventative strategies.

## Methods

### Source population

Kaiser Permanente Northern California (KPNC) is a large, integrated health care delivery system that currently provides comprehensive inpatient and outpatient care for > 4.1 million members in the greater San Francisco Bay Area. Its membership is highly diverse and representative of the local surrounding and statewide population [23], with nearly all aspects of care captured through KPNC’s electronic medical record system.

This study was approved by the institutional review boards at KPNC and University of California, San Francisco (#16–20030). We obtained a waiver of informed consent given the nature of the study.

### Study sample

We identified all adult (age ≥18 years) KPNC members who developed AKI-D or who initiated chronic hemodialysis between January 2009 and September 2015 and who had ≥12 consecutive months of health plan membership and pharmacy benefits before initiation of renal replacement therapy (RRT) in order to ensure adequate capture of relevant comorbidities and prescription medication use. We defined AKI-D as receipt of RRT during hospitalization in the absence of any pre-admission RRT (dialysis or transplant). Inpatient RRT included receipt of peritoneal dialysis, hemodialysis, and hemofiltration that were identified using *International Classification of Diseases, Ninth Revision* (ICD-9) procedure codes (54.98, 39.95) and *Current Procedural Terminology* codes (90,935, 90,937, 90,945, 90,947, 90,999). We previously demonstrated the accuracy of these codes to identify AKI-D across the spectrum of pre-admission estimated glomerular filtration rate (eGFR) based on adjudication of medical records by a board-certified nephrologist in a random sample of 100 patients (positive predictive value 94%) [24]. We excluded one patient with pre-hospitalization eGFR > 150 mL/min/1.73m^2^ because of concerns about the accuracy of the value. Patients initiating chronic hemodialysis without preceding AKI-D were ascertained through a comprehensive health system ESRD Treatment Registry [21, 22, 25]. For incident ESRD patients who did not have AKI-D, we studied only hemodialysis patients because risk factors for early death and CVD events may differ according to ESRD treatment modality, and because hemodialysis is the most common initial modality in the U.S. [8].

### Predictor variable

The two primary comparisons were 1) between patients with incident ESRD due to non-recovery from AKI-D versus incident ESRD patients who did not have AKI-D and 2) between AKI-D patients who did versus who did not recover adequate kidney function to discontinue dialysis. Recovery from AKI-D was defined as being alive and no longer needing RRT for ≥4 weeks at 90 days after initiation of acute RRT. We required that patients remain alive for ≥4 weeks to reduce potential misclassification due to withdrawal of care. To be comprehensive, recovery could occur during the index hospitalization or in the outpatient setting after hospital discharge. We used recovery status at 90 days, as patients are conventionally considered to have ESRD using this cutoff [26].

### Follow-up and outcome variables

We focused on short-term clinical outcomes of AKI-D because we *a priori* hypothesized that the effect of AKI-D would gradually fade over time, consistent with findings regarding the potential effect of acute kidney injury on other outcomes [16]. Starting 90 days after RRT initiation, patients were censored at health plan disenrollment or death up to 365 days. We excluded patients censored before 90 days post-RRT initiation because 90-day survival was necessary to ascertain recovery from AKI-D, as well as patients with hospitalizations ≥90 days after acute RRT initiation.

Primary clinical outcomes included all-cause death, heart failure, acute coronary syndrome (ACS), and acute ischemic stroke or transient ischemic attack (TIA) occurring between 90 days and 455 days (i.e., up to one year later) after RRT initiation using validated diagnosis codes and algorithms with high positive predictive values based on data found in comprehensive health plan electronic medical records (codes available upon request) [27, 28]. Vital status was based on comprehensive information from health plan administrative and hospital discharge databases, member proxy reporting, Social Security Administration vital status files, and California state death certificate information [27, 29].

### Covariates

We relied primarily on electronic health record data that were standardized and linked at the patient-level in the Kaiser Permanente Virtual Data Warehouse [21, 28, 30–32]. Demographic and lifestyle characteristics included age, gender, self-reported race/ethnicity, and tobacco use. Relevant pre-admission comorbidities (heart failure, coronary disease, ischemic stroke, peripheral artery disease, atrial fibrillation, mitral/aortic valvular disease, hypertension, diabetes mellitus, dyslipidemia, prior hospitalized bleed, thyroid disease, cirrhosis, lung disease, dementia, and depression) were defined using validated diagnostic or procedure codes [33]. We ascertained outpatient body mass index and systolic blood pressure, as well as relevant outpatient laboratory test results (eGFR using the CKD-EPI equation, urine dipstick proteinuria, high-density lipoprotein, low-density lipoprotein, and hemoglobin levels) and receipt of medications (angiotensin converting enzyme inhibitors, angiotensin II receptor blockers, beta blockers, calcium channel blockers, diuretics, aldosterone receptor antagonists, alpha blockers, antiarrhythmic agents, nitrates, other vasodilators, non-aspirin antiplatelet agents, low-molecular-weight heparin, statins, other lipid-lowering agents, anti-diabetic agents, and non-steroidal anti-inflammatory drugs).

### Statistical approach

All analyses were conducted using SAS, version 9.3 (Cary, N.C.). Baseline characteristics were compared across exposure categories using ANOVA for continuous variables and χ^2^ tests for categorical variables.

We calculated crude incidence rates and 95% confidence intervals for outcomes by exposure group. After confirming no violation of the proportional hazards assumption using visual examination of Kaplan-Meier curves and ln(−ln) plots and assessment of Schoenfeld residuals, we conducted Cox regression models for each outcome of interest with adjustment for demographics, tobacco use, comorbidities, vital signs, and medication use.

We also conducted sensitivity analyses using AKI-D patients with a pre-existing eGFR > 24 ml/min/1.73 m^2^, which was the 75th percentile threshold for this subset of our cohort (ESRD due to AKI-D *n* = 434, ESRD not due to AKI-D *n* = 3772, recovered AKI-D *n* = 942).

## Results

### Baseline characteristics

We initially identified 13,213 hospitalized patients who received inpatient RRT and 6414 patients who initiated chronic hemodialysis as outpatients between January 2009 and September 2015. After applying exclusion criteria, our final sample included 1865 patients with incident ESRD due to AKI-D (i.e., non-recovery from AKI-D), 3772 patients with incident ESRD not due to AKI-D, and 1347 AKI-D patients who recovered within 90 days after RRT initiation.

Compared to incident ESRD patients without AKI-D, patients with incident ESRD due to AKI-D were older, more likely to be white, and generally had a higher burden of baseline CVD (Table 1). Compared to AKI-D patients who did not recover, AKI-D patients who recovered were younger, more likely to be white, had higher baseline eGFR, and were less likely to have pre-existing heart failure and other CVD, hypertension, diabetes, or dyslipidemia (Table 1).

**Table 1:** Baseline characteristics of study cohort. Baseline was defined as pre-hospitalization for patients with dialysis-requiring acute kidney injury and at the time of dialysis initiation for patients without dialysis-requiring acute kidney injury

	Overall	ESRD Not Due to AKI-D	ESRD Due to AKI-D	Recovered AKI-D	
Characteristic	(*n* = 6984)	(*n* = 3772)	(*n* = 1865)	(*n* = 1347)	*P*-Value
Age, years, mean (SD)	64.9 (14.0)	64.8 (13.9)	66.7 (13.5)	62.9 (14.7)	< 0.001
Female Gender, n (%)	3007 (43.1)	1663 (44.1)	806 (43.2)	538 (39.9)	< 0.05
Self-reported Race, n (%)					
White/European	2787 (39.9)	1295 (34.3)	763 (40.9)	729 (54.1)	< 0.001
Black/African American	1128 (16.2)	638 (16.9)	337 (18.1)	153 (11.4)	
Asian/Pacific Islander	1397 (20.0)	911 (24.2)	322 (17.3)	164 (12.2)	
Other/Unknown	1672 (23.9)	928 (24.6)	443 (23.8)	301 (22.3)	
Hispanic ethnicity, n (%)	1412 (20.2)	821 (21.8)	363 (19.5)	228 (16.9)	< 0.001
Smoking status, n (%)					< 0.001
Current Smoker	529 (7.6)	228 (6.0)	149 (8.0)	152 (11.3)	
Former smoker	2926 (41.9)	1580 (41.9)	809 (43.4)	537 (39.9)	
Nonsmoker	3529 (50.5)	1964 (52.1)	907 (48.6)	658 (48.8)	
Cardiovascular history, n (%)					
Acute myocardial infarction	373 (5.3)	184 (4.9)	135 (7.2)	54 (4.0)	< 0.001
Coronary artery bypass graft surgery	111 (1.6)	54 (1.4)	36 (1.9)	21 (1.6)	0.37
Percutaneous coronary intervention	261 (3.7)	128 (3.4)	89 (4.8)	44 (3.3)	< 0.05
Heart failure	2162 (31.0)	1092 (29.0)	757 (40.6)	313 (23.2)	< 0.001
Ischemic stroke or TIA	323 (4.6)	189 (5.0)	88 (4.7)	46 (3.4)	0.06
Peripheral artery disease	369 (5.3)	264 (7.0)	76 (4.1)	29 (2.2)	< 0.001
Mitral and/or aortic valvular disease	721 (10.3)	313 (8.3)	247 (13.2)	161 (12.0)	< 0.001
Atrial flutter or fibrillation	943 (13.5)	408 (10.8)	326 (17.5)	209 (15.5)	< 0.001
Venous thromboembolism	128 (1.8)	53 (1.4)	37 (2.0)	38 (2.8)	< 0.01
Other medical history, n (%)					
Diabetes mellitus	4494 (64.3)	2544 (67.4)	1284 (68.8)	666 (49.4)	< 0.001
Hypertension	6409 (91.8)	3688 (97.8)	1721 (92.3)	1000 (74.2)	< 0.001
Dyslipidemia	5872 (84.1)	3376 (89.5)	1566 (84.0)	930 (69.0)	< 0.001
Body mass index, kg/m^2^, mean (SD)	30.4 (7.7)	29.8 (7.2)	30.8 (8.0)	31.6 (8.5)	< 0.001
Systolic blood pressure, mmHg, mean (SD)	134.0 (21.5)	135.8 (20.0)	135.9 (24.1)	126.0 (19.8)	< 0.001
Baseline medication use, n (%)					
Angiotensin converting enzyme inhibitor	1587 (22.7)	684 (18.1)	441 (23.6)	462 (34.3)	< 0.001
Angiotensin II receptor blocker	1110 (15.9)	615 (16.3)	290 (15.5)	205 (15.2)	0.58
Beta blocker	4403 (63.0)	2590 (68.7)	1179 (63.2)	634 (47.1)	< 0.001
Calcium channel blocker	3959 (56.7)	2574 (68.2)	1007 (54.0)	378 (28.1)	< 0.001
Diuretic	4738 (67.8)	2746 (72.8)	1300 (69.7)	692 (51.4)	< 0.001
Alpha blocker	1919 (27.5)	1202 (31.9)	504 (27.0)	213 (15.8)	< 0.001
Aldosterone receptor antagonist	272 (3.9)	68 (1.8)	101 (5.4)	103 (7.6)	< 0.001
Nitrates	1142 (16.4)	626 (16.6)	396 (21.2)	120 (8.9)	< 0.001
Vasodilators	1976 (28.3)	1248 (33.1)	570 (30.6)	158 (11.7)	< 0.001
Antiarrhythmic drug	156 (2.2)	55 (1.5)	54 (2.9)	47 (3.5)	< 0.001
Statin	4561 (65.3)	2647 (70.2)	1202 (64.5)	712 (52.9)	< 0.001
Other lipid-lowering agent	513 (7.3)	294 (7.8)	119 (6.4)	100 (7.4)	0.16
Non-aspirin antiplatelet agent	526 (7.5)	284 (7.5)	160 (8.6)	82 (6.1)	< 0.05
Low molecular weight heparin	94 (1.3)	28 (0.7)	29 (1.6)	37 (2.7)	< 0.001
Non-steroidal anti-inflammatory drug	238 (3.4)	41 (1.1)	71 (3.8)	126 (9.4)	< 0.001
Diabetic therapy	2820 (40.4)	1588 (42.1)	796 (42.7)	436 (32.4)	< 0.001
Baseline laboratory values					
CKD-EPI eGFR, mL/min/1.73 m^2^					
Median (interquartile range)	11.7 (8.4–20.0)	9.8 (7.6–12.4)	14.2 (9.5–24.1)	51.4 (30.6–77.0)	< 0.001
Missing, n (%)	392 (5.6)	57 (1.5)	130 (7.0)	205 (15.2)	
Urine dipstick proteinuria					< 0.001
Negative/Trace	700 (10.0)	218 (5.8)	189 (10.1)	293 (21.8)	
1+	999 (14.3)	515 (13.7)	279 (15.0)	205 (15.2)	
≥2+	4246 (60.8)	2889 (76.6)	1109 (59.5)	248 (18.4)	
Unknown	1039 (14.9)	150 (4.0)	288 (15.4)	601 (44.6)	

### Outcomes between ESRD due to AKI-D vs. ESRD not due to AKI-D

In unadjusted analyses, patients with incident ESRD due to AKI-D had higher all-cause death rates than patients with incident ESRD who did not experience AKI-D (Table 2). Patients with incident ESRD due to AKI-D also had significantly higher crude heart failure hospitalization and ACS rates but no significant differences in stroke/TIA.

**Table 2:** Crude rates of all-cause death and cardiovascular outcomes after dialysis initiation, stratified by end-stage renal disease (ESRD) and dialysis-requiring acute kidney injury (AKI-D) status

Outcome	Rate per 100 person-years (95% Confidence Interval)	P-value
All-Cause Death		
ESRD due to AKI-D	23.58 (18.06–30.78)	Ref
ESRD not due to AKI-D	9.51 (8.50–10.65)	< 0.0001
Recovered AKI-D	17.17 (12.78–23.05)	0.0004
Hearth Failure Hospitalization		
ESRD due to AKI-D	8.17 (5.09–13.11)	Ref
ESRD not due to AKI-D	3.00 (2.45–3.67)	< 0.0001
Recovered AKI-D	7.68 (4.66–12.65)	0.5858
Acute Coronary Syndrome		
ESRD due to AKI-D	5.14 (3.17–8.33)	Ref
ESRD not due to AKI-D	3.45 (2.86–4.17)	0.0081
Recovered AKI-D	2.21 (1.18–4.15)	0.0003
Stroke or Transient Ischemic Attack		
ESRD due to AKI-D	3.53 (2.02–6.18)	Ref
ESRD not due to AKI-D	2.68 (2.16–3.32)	0.1168
Recovered AKI-D	1.19 (0.54–2.65)	0.0005

**Fig. 1:**
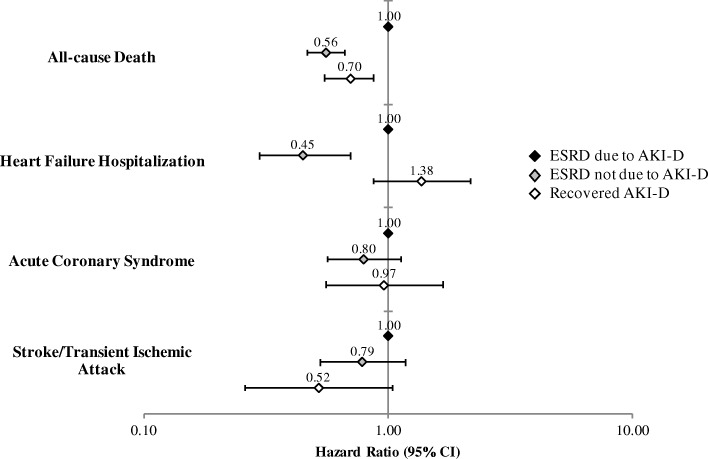
Multivariable association of all-cause mortality and cardiovascular events after dialysis initiation among patients with incident end-stage renal disease (ESRD) due to dialysis-requiring acute kidney injury (AKI-D), patients with incident ESRD not due to AKI-D, and patients with AKI-D who recovered. Models are adjusted for the following baseline covariates: age, gender, race, Hispanic ethnicity, smoking status, acute myocardial infarction, heart failure, ischemic stroke or transient ischemic attack, peripheral artery disease, mitral and/or aortic valvular disease, atrial flutter or fibrillation, venous thromboembolism, other thromboembolic events, coronary artery bypass surgery, percutaneous coronary intervention, diabetes mellitus, hypertension, dyslipidemia, hyperthyroidism, hypothyroidism, cirrhosis, chronic lung disease, diagnosed dementia, diagnosed depression, hospitalized bleed, body mass index, systolic blood pressure, eGFR, dipstick proteinuria, hemoglobin level, HDL cholesterol level, LDL cholesterol level, and pre-admission receipt of the following medications: ACE inhibitor, angiotensin II receptor blocker, antiarrhythmic drug, beta blocker, calcium channel blocker, diuretic, alpha blocker, any anti-hypertensive agent, aldosterone receptor antagonist, nitrates, vasodilators, statin, other lipid-lowering agent, non-aspirin antiplatelet agent, low molecular weight heparin, non-steroidal anti-inflammatory drug, and diabetic therapy

In multivariable analyses, compared to patients with incident ESRD due to AKI-D, those with incident ESRD not due to AKI-D had a significantly lower adjusted rate of all-cause death (adjusted hazard ratio [aHR] 0.56, 95% CI 0.47–0.67) (Fig. 1). After adjustment for potential confounders, ESRD not due to AKI-D was also associated with a lower adjusted rate of heart failure hospitalization (aHR 0.45, 0.30–0.70) but not ACS or stroke/TIA (Fig. 1).

In sensitivity analyses excluding AKI-D patients with pre-existing eGFR ≤24 ml/min/1.73 m^2^, we obtained similar crude and adjusted results for death (aHR 0.43, 0.28–0.67) and heart failure hospitalization (aHR 0.61, 0.27–1.37).

### Outcomes between recovery vs. non-recovery from AKI-D

Patients who recovered from AKI-D had lower crude rates of all-cause death, ACS, and stroke/TIA compared to those who did not recover (Table 2). In multivariable analyses, recovery from AKI-D was independently associated with lower mortality (aHR 0.70, 0.55–0.88) compared with non-recovery (Fig. 1). However, there were no significant adjusted differences in rates of heart failure, ACS, or stroke/TIA (Fig. 1). We obtained similar crude and adjusted results in our sensitivity analyses (aHR for death 0.59, 0.44–0.80).

## Discussion

Healthy People 2020 includes as key objectives to reduce all-cause and CVD-related mortality in ESRD patients [34]. While the U.S. Renal Data System tracks long-term mortality in ESRD patients, ascertainment of both early death and CVD events, as well as understanding potentially modifiable risk factors, has been much more challenging. Our study based within a large, community-based population helps to fill this important knowledge gap.

Our study has several key findings. First, a large fraction (one third) of incident ESRD patients in our cohort developed ESRD via AKI-D. Because we censored patients who died within 90 days of RRT initiation, one caveat is that the numbers of incident ESRD patients in both the AKI-D and non-AKI-D groups were reduced. It can also be clinically challenging to determine whether patients with chronic kidney disease who initiate dialysis during a hospitalization do so because of progression of underlying disease versus superimposed acute injury. However, prior single-center and VA data have reported similar proportions of incident ESRD patients initiating dialysis in the hospital [35–37]. Therefore, our finding that patients with incident ESRD due to AKI-D have significantly higher adjusted all-cause death rates compared to incident ESRD patients without AKI-D indicates that the large number of patients who reach ESRD via AKI-D may explain, in part, the high early mortality rate in the overall ESRD population.

Second, we found that patients with incident ESRD due to AKI-D have higher rates of heart failure hospitalization after RRT initiation than ESRD patients without AKI-D. These findings extend prior observations that patients with abrupt decline in renal function before ESRD have higher rates of heart failure events in the three months before dialysis initiation compared to those who did not experience an abrupt decline [20], and patients who initiate dialysis due to volume overload have higher mortality rates [38]. In our cohort, patients with ESRD due to AKI-D were more likely to have pre-existing heart failure compared to patients with ESRD not due to AKI-D. Taken together, these data suggest that the higher mortality rates among those who experienced AKI-D leading to ESRD may be driven, in part, by a greater burden of heart failure. This finding suggests that better fluid management and more systematic use of evidence-based therapies for heart failure may potentially improve outcomes in this vulnerable population.

Third, our finding that AKI-D patients who recovered had lower adjusted mortality compared to those who did not recover highlights yet another reason we urgently need to devise treatment strategies to enhance or hasten recovery from AKI-D. Most prior studies evaluating long-term sequelae after recovery from AKI-D compared AKI-D patients to matched controls without AKI-D [13, 14], while we were able to distinguish outcome differences between those who did and did not recover. Although one small study reported that patients who recovered after AKI-D had lower 2-year mortality compared to patients who did not recover after AKI-D, our cohort was substantially larger and able to account for a larger set of potential confounders [39].

Finally, although we hypothesized that patients who recovered after AKI-D would have better outcomes, we found that even those who are able to discontinue dialysis remain at high risk for cardiovascular events, particularly hospitalizations for heart failure. We examined early outcomes after RRT initiation because mortality is particularly high during this time [8] and because AKI-D may be linked to elevated short-term CVD risk [16]. Several studies in the general population have suggested a transient increase in risk of stroke or myocardial infarction after an episode of infection or systemic inflammation [40, 41], and another study found that septicemia is a CVD risk factor in ESRD patients [42]. However, we did not find significant differences in outcomes that may be related to arterial plaque rupture or occlusion (e.g., ACS, stroke/TIA). Instead, AKI-D leading to ESRD was most strongly associated with all-cause death and excess heart failure events, which is also linked to aberrations in multiple signaling pathways related to inflammation, fluid overload, myocardial injury, and ventricular stress [43, 44].

Strengths of our study included the large number of AKI-D cases, which allowed us to examine the impact of renal recovery on clinical outcomes. In contrast to the majority of studies examining highly selected populations (e.g., post-operative acute kidney injury, contrast-induced nephropathy) [9], our study included a broad mix of AKI-D etiologies, which increases generalizability. We were also able to examine the pre-ESRD disease course (prior medication use, medical history, and other clinical features) to characterize incident ESRD subgroups and add important new insights on the transition to ESRD via AKI-D as well as the impact of recovery from AKI-D on CVD outcomes [8, 37, 39, 45, 46]. Furthermore, our data were much more rigorous than data collected via the Medical Evidence Report that is used for registration into the national U.S. Renal Data System [47–50].

Limitations include that some patients classified in our study as having AKI-D may have initiated dialysis as inpatients because of progression of advanced chronic kidney disease rather than true AKI-D. However, for the comparison of patients with ESRD due to vs. not due to AKI-D, this type of misclassification would bias results toward the null, which bolsters our confidence in the differences we detected. We did not have details on inpatient dialysis prescriptions (e.g., modality, dose, or anticoagulation use), or etiology of AKI-D, including underlying cardiorenal physiology. Only insured patients from Northern California enrolled in an integrated health care delivery system were included, but the KPNC membership is highly representative of the local surrounding and statewide population, which argues for greater generalizability [23]. While we relied on electronic medical records for key data elements, our data sources are much more comprehensive than administrative databases and include clinical data elements (instead of primarily relying on diagnostic or procedural codes) [21, 28, 30–32]. Because we wanted to explore how recovery from AKI-D influences outcomes, we were only able to compare outcomes that occurred between 90 days after RRT initiation and up to 1 year thereafter. Conditioning on survival to 90 days (in all three subgroups examined) may introduce immortal time bias, but this potential bias would likely decrease the observed effect size because the sickest AKI-D patients in the non-recovery group who died early would be excluded. We also had to exclude a small number (*n* = 182) of patients who were hospitalized for ≥ 90 days during their AKI-D hospitalization, but again these patients were likely the sickest AKI-D patients who would have been at highest risk for early death and CVD events. However, our design did preclude addressing potential differences in death and CVD event rates during the first 90 days, which may be higher as compared to the subsequent 1-year follow-up period.

## Conclusions

In conclusion, we found that patients who transition to ESRD via AKI-D are at higher risk for short-term death and heart failure hospitalization compared to those who start chronic dialysis without experiencing AKI-D. Our findings indicate that the high short-term mortality in incident ESRD patients may be explained, at least in part, by the subset of patients who developed ESRD via AKI-D. Furthermore, our findings suggest that this subset of incident ESRD patients may have worse outcomes because of pre-existing heart failure. Recovery from AKI-D was also independently associated with lower short-term mortality. Collectively, these findings suggest that aggressive surveillance for and medical management of heart failure in ESRD patients may be a potential strategy for reducing early mortality after AKI-D. Further studies are warranted to evaluate whether promotion of renal recovery from AKI-D and systematic surveillance and intervention for heart failure can improve outcomes in this vulnerable patient population.

## Abbreviations

ACS: acute coronary syndrome
aHR: adjusted hazard ratio
AKI-D: dialysis-requiring acute kidney injury
CVD: cardiovascular disease
eGFR: estimated glomerular filtration rate
ESRD: end-stage renal disease
ICD-9: International Classification of Diseases, Ninth Revision
KPNC: Kaiser Permanente Northern California
RRT: renal replacement therapy
TIA: transient ischemic attack
